# Potential of dried plasma spot: a comparative study of plasma biomarker quantification using NULISA

**DOI:** 10.1002/alz70856_106865

**Published:** 2026-01-08

**Authors:** You‐Rim Lee, Xuemei Zeng, Jeremy M. Gu, Marissa F Farinas, Julia K. Kofler, Dana L Tudorascu, C. Elizabeth Shaaban, Jennifer H Lingler, Tharick A Pascoal, William E Klunk, Victor L. Villemagne, Sarah B Berman, Robert Sweet, Beth E. Snitz, Milos D. Ikonomovic, M. Ilyas Kamboh, Oscar L Lopez, Ann D Cohen, Thomas K Karikari

**Affiliations:** ^1^ University of Pittsburgh School of Medicine, Pittsburgh, PA, USA; ^2^ Department of Psychiatry, University of Pittsburgh, Pittsburgh, PA, USA; ^3^ University of Pittsburgh Alzheimer's Disease Research Center (ADRC), Pittsburgh, PA, USA; ^4^ Departments of Psychiatry and Neurology, University of Pittsburgh School of Medicine, Pittsburgh, PA, USA; ^5^ The University of Pittsburgh, Pittsburgh, PA, USA; ^6^ UPMC, Pittsburgh, PA, USA; ^7^ University of Pittsburgh, Pittsburgh, PA, USA

## Abstract

**Background:**

Efforts to diagnose Alzheimer's disease (AD) through blood tests have identified many promising biomarkers. However, traditional plasma collection via venipuncture requires a medical professional and cold‐chain transport, making it unsuitable for remote or home‐based testing. Dried plasma spots (DPS) allow self‐sampling and easier storage/transport but only collect a small amount of plasma. The Nucleic Acid‐Linked Immuno‐Sandwich Assay (NULISA) is an innovative platform that quantifies biomarkers from small plasma. This study compares DPS and traditional plasma using NULISA to evaluate DPS's potential for AD biomarkers.

**Method:**

Venous blood from 85 paricipants at the University of Pittsburgh Alzheimer's Disease Research Center, Connectomics in Brain Aging (CoBrA), and INflammation ROles in Aging and Alzheimer's disease Study (INROAADS). DPS samples were created by applying blood to Telimmune Duo Plasma Prep Cards. Traditional plasma samples were collected through centrifugation. Alamar sample diluent was used to extract proteins from DPS. NULISAseq CNS Panel was performed on an Argo HT following the manufacturer's instructions. The reproducibility of assays was evaluated using a Sample Control run in triplicate across runs. Spearman correlation assessed the concordance between DPS and traditional plasma samples.

**Result:**

The NULISAseq CNS panel quantified 127 targets with high reproducibility, showing median intra‐ and inter‐plate coefficients of variation <10%. In DPS samples, 104 targets (82%) were detected above the limit of detection (LOD) in >50% of samples, compared to 123 (97%) in plasma. Median sample detectability was 82.7% for DPS and 96.1% for plasma. Among the classical AD biomarkers, NFL, GFAP, MAPT, *p*‐tau181, *p*‐tau217, and *p*‐tau231 showed high detectability (71% to 99%), while Aβ38, Aβ40, and Aβ42 had low detectability (29% to 35%). A moderate correlation was observed between DPS and plasma samples for these biomarkers, with correlation coefficients ranging from 0.197 to 0.616. Across the whole panel, the median correlation coefficient was 0.369, with NEFH (0.831), CHIT1 (0.829), and IL5 (0.783) showing the highest correlation.

**Conclusion:**

Our results support the potential of DPS for remote and home‐based testing, enabled by technologies like NULISA that quantify biomarkers from low plasma volumes. Further optimization of DPS methods is needed to improve concordance with classical plasma samples.

